# Intra-host *Trypanosoma cruzi* strain dynamics shape disease progression: the missing link in Chagas disease pathogenesis

**DOI:** 10.1128/spectrum.04236-22

**Published:** 2023-09-05

**Authors:** Eric Dumonteil, Hans Desale, Weihong Tu, Nora Hernandez-Cuevas, Monica Shroyer, Kelly Goff, Preston A. Marx, Claudia Herrera

**Affiliations:** 1 Department of Tropical Medicine, School of Public Health and Tropical Medicine, and Vector-Borne and Infectious Disease Research Center, Tulane University, New Orleans, Louisiana, USA; 2 Laboratorio de Parasitologia, Centro de Investigaciones Regionales “Dr. Hideyo Noguchi”, Universidad Autonoma de Yucatan, Merida, Yucatan, Mexico; 3 Division of Veterinary Medicine, Tulane National Primate Research Center, Tulane University, Covington, Louisiana, USA; 4 Division of Microbiology, Tulane National Primate Research Center, Tulane University, Covington, Louisiana, USA; Institut de Recherche pour le Développement, Montpellier, France

**Keywords:** strain interactions, host-parasite relationship, intracellular parasites, competition, pathogenesis

## Abstract

Chronic Chagasic cardiomyopathy develops years after infection in 20–40% of patients, but disease progression is poorly understood. Here, we assessed *Trypanosoma cruzi* parasite dynamics and pathogenesis over a 2.5-year period in naturally infected rhesus macaques. Individuals with better control of parasitemia were infected with a greater diversity of parasite strains compared to those with increasing parasitemia over time. Also, the *in vivo* parasite multiplication rate decreased with increasing parasite diversity, suggesting competition among strains or a stronger immune response in multiple infections. Significant differences in electrocardiographic (ECG) profiles were observed in Chagasic macaques compared to uninfected controls, suggesting early conduction defects, and changes in ECG patterns over time were observed only in macaques with increasing parasitemia and lower parasite diversity. Disease progression was also associated with plasma fibronectin degradation, which may serve as a biomarker. These data provide a novel framework for the understanding of Chagas disease pathogenesis, with parasite diversity shaping disease progression.

**IMPORTANCE:**

Chagas disease progression remains poorly understood, and patients at increased risk of developing severe cardiac disease cannot be distinguished from those who may remain asymptomatic. Monitoring of *Trypanosoma cruzi* strain dynamics and pathogenesis over 2–3 years in naturally infected macaques shows that increasing parasite diversity in hosts is detrimental to parasite multiplication and Chagasic cardiomyopathy disease progression. This provides a novel framework for the understanding of Chagas disease pathogenesis.

## INTRODUCTION

Chagas disease is a neglected tropical disease caused by *Trypanosoma cruzi* parasites, with a disease burden of 0.55 million disability-adjusted life years lost, 10,600 annual deaths, and an estimated $7.2 billion in annual economic losses (1). Its main clinical manifestation is chronic Chagasic cardiomyopathy, which develops many years after the initial infection in 20–40% of patients, while digestive manifestations such as mega-esophagous or megacolon may be observed in about 10% of patients (2). Despite the severe morbidity associated with disease progression, Chagas disease remains often underdiagnosed and has substandard access to care for patients.

A major issue is that disease progression is still poorly understood, and patients at an increased risk of developing severe disease cannot be distinguished from those who may remain in the asymptomatic chronic stage, making any prognosis uncertain. Pathogenesis has been associated with immune exhaustion, allowing parasite persistence in host tissues (3, 4), providing the rationale to target the parasite through drugs or therapeutic vaccines (2). However, the efficacy of benznidazole treatment is variable, and while it can consistently decrease parasite burden, it can also delay the appearance of new electrocardiographic (ECG) abnormalities and the progression to more severe Kuschnir group of cardiac disease in patients treated early (5, 6), but it fails to stop or delay the progression of fibrotic heart disease in symptomatic patients (7). However, disease progression and response to treatment are difficult to monitor, due in part to the need for long-term follow-up and the lack of precise indicators. For example, seronegativization, which may be considered the gold standard for treatment response, takes many years after treatment to be observed (8, 9).

Several alternative biomarkers of disease progression and response to treatment have been proposed. For example, in Chagasic mice, serum levels of transforming growth factor β, platelet-derived growth factor, and connective tissue growth factor correlate well with cardiac fibrosis and cardiac dysfunction (10). Plasma fibronectin degradation has also been proposed as a biomarker of disease progression and response to treatment in both patients and mouse models (11 – 14). Nonetheless, the prognosis value of these biomarkers remains to be investigated.

Adding to this complexity, parasite genetic diversity has also been suggested to play a role in Chagas disease pathogenesis. Indeed, *T. cruzi* parasites are genetically very diverse, and the species is currently divided into seven major lineages or discrete typing units (DTUs) named TcI to TcVI and TcBat (15). While clear differences in infectivity, virulence, or drug susceptibility can be observed among parasite strains *in vitro* or in animal models (16 – 20), marked associations of parasite DTUs with clinical manifestations in patients have been difficult to establish (21 – 25). In addition, the use of genotyping methods of increased sensitivity is providing growing evidence that a large proportion of infections in both triatomine vectors (26 – 28) and mammalian hosts (29 – 33) are in fact infections with multiple parasite strains or DTUs. For example, mixed infections with TcI and non-TcI parasites were detected in 20.6% (7/34) of pregnant women in Mexico, 38.1% (8/21) in Honduras, 43.7% (6/16) in Argentina (30), and in 33% (2/6) of US patients from Texas (34). Another study found that 47% (8/17) of Chagasic patients from Yucatan, Mexico, were infected with different mixtures of TcI, TcII, TcV, and TcVI parasites (31). The implications of such multiple infections on disease progression and clinical manifestations are unclear, but interactions among strains are likely to occur, which will modulate selection pressures on both hosts and parasite strains (35 – 38). For example, experimental co-infection with two strains of *Trypanosoma brucei* in mice resulted in the suppression of individual strains and increased host survival (39). Accordingly, experimental co-infections with *T. cruzi* in mice can also result in differences in infection progression (40 – 42), suggesting interactions among strains, but these favor either the host or the parasite, depending on specific strain combinations, making generalization of these results difficult. Theoretical approaches have helped conceptualize these possible outcomes, such as a more severe disease caused by multiple pathogen strains when their “super-infection” is able to better evade or overcome the immune system. Conversely, less severe disease may occur if competition among strains or cross-immunity leads to reduced pathogen growth. However, there is a lack of empirical data to assess the relevance of these potential situations and outcomes (35), particularly in the context of natural infections with *T. cruzi*.

Therefore, our objective was to assess *T. cruzi* parasite strain dynamics and disease progression in a cohort of naturally infected rhesus macaques, which closely mimic human infection (43). There is indeed extensive zoonotic circulation of *T. cruzi* in the southern USA (44), with occasional spill-over infections in captive macaques (45) with *T. cruzi* strains that are similar to those circulating in local *Triatoma sanguisuga* vectors and mammalian hosts in Louisiana (26, 29, 32, 33, 46, 47). We measured parasite dynamics and clinical disease progression over a 2.5-year period in macaques that had been infected for 1–6 years to shed light for the first time on a unique interplay between multiple parasite strains and clinical profile during the chronic phase and provide a new framework for understanding Chagas disease progression.

## RESULTS

### Parasite burden in a cohort of naturally infected rhesus macaques

**Fig 1:**
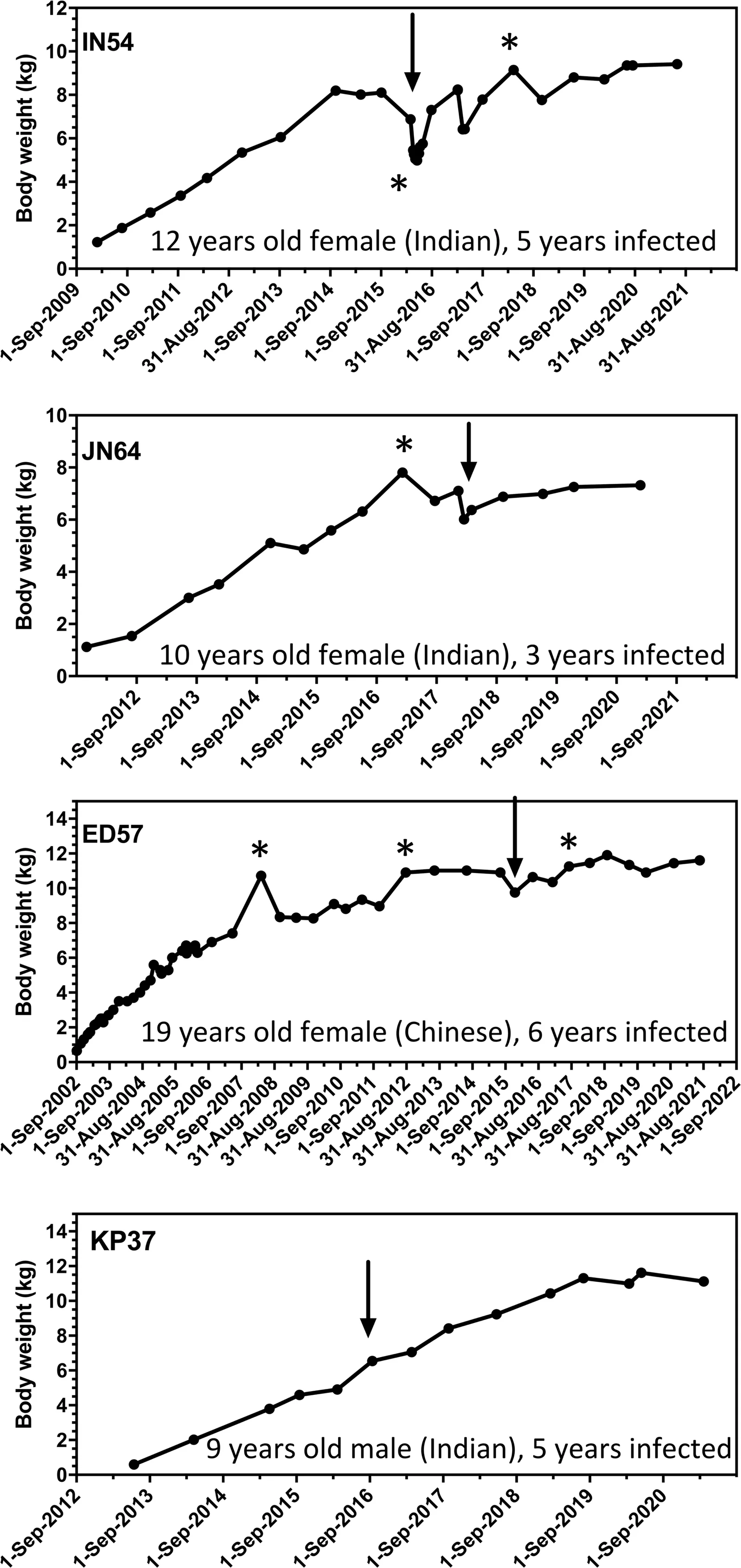
Body weight history. Body weight history of representative Chagasic macaques. Vertical arrows indicate the time when positive *T. cruzi* serology was first detected. * indicate pregnancies, which may explain some body weight increases. Note some transient body weight loss detected in some animals around the time of infection.

We followed a cohort of rhesus macaques with a natural *T. cruzi* infection. These were initially screened as seropositive for *T. cruzi* during routine health examinations of a breeding colony, and infection was confirmed through additional serological testing (Stat-Pak rapid test) and PCR. Their average age was 10.7 ± 0.7 years old (range four to 19 years), and they had been infected for 4.1 ± 0.3 years at the start of the follow-up (range one to 6 years) (Table S1). The cohort included five macaques of Chinese ancestry and 27 of Indian ancestry. Some animals, but not all, presented a transient loss in body weight around the time seroconversion was detected, which may reflect the acute phase of *T. cruzi* infection (Fig. 1).

**Fig 2:**
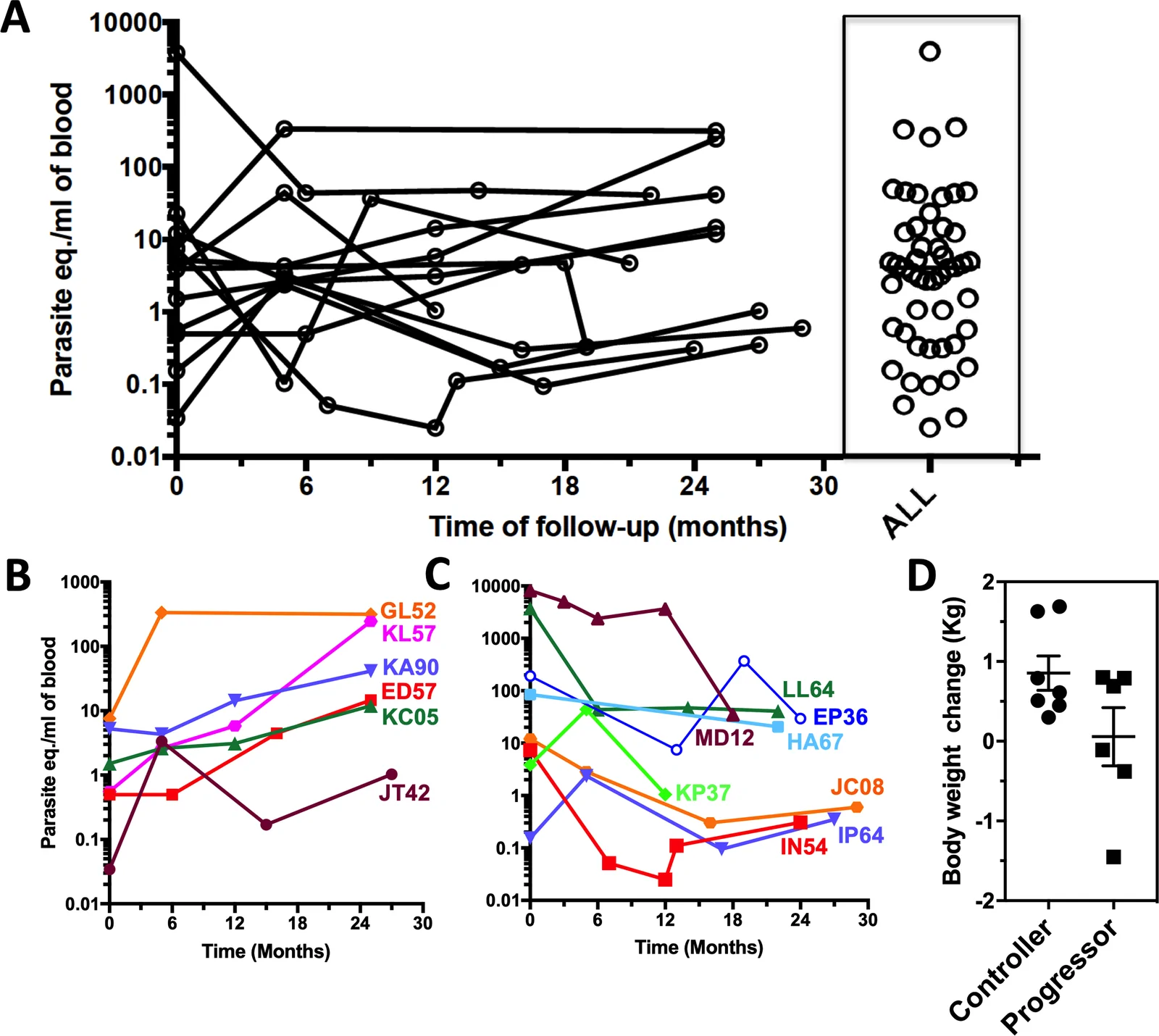
Blood parasite burden. Blood parasite burden was measured by qPCR over time. (**A**) Time course of parasitemia during 24–30 months of follow-up in Chagasic rhesus macaques. (**B**) Time course of parasitemia in individual macaques with increasing parasitemia (“progressors”). (**C**) Time course of parasitemia in individual macaques with decreasing parasitemia (“controllers”). Symbols and lines are color-coded for each macaque, with their ID indicated in the graph in panels B and C. (**D**) Body weight change at the time of infection in progressor and controller macaques. Body weight loss tended to be more pronounced in progressor macaques, although this did not reach statistical significance (*P* = 0.076).

Quantification of *T. cruzi* parasitemia by qPCR indicated an average parasite burden of 8.1 ± 1.5 parasite eq./mL of blood that was overall stable over the 24–30 months of follow-up (Fig. 2A). However, analysis of the changes over time in parasite burden in individual macaques revealed two major patterns, with some animals presenting a slow decrease in parasitemia over time, suggesting some control of the infection, which were referred to as “controllers” (Fig. 2B), while others presented a gradual increase in parasitemia, suggestive of disease progression, which were referred to as “progressors” (Fig. 2C). Interestingly, macaques with progressive disease tended to have had more pronounced body weight loss at the time of infection compared with those with better control of the infection, suggesting a more severe acute phase, although this did not reach statistical significance (Fig. 2D, *P* = 0.076).

### Parasite genotyping and strain dynamics

**Fig 3:**
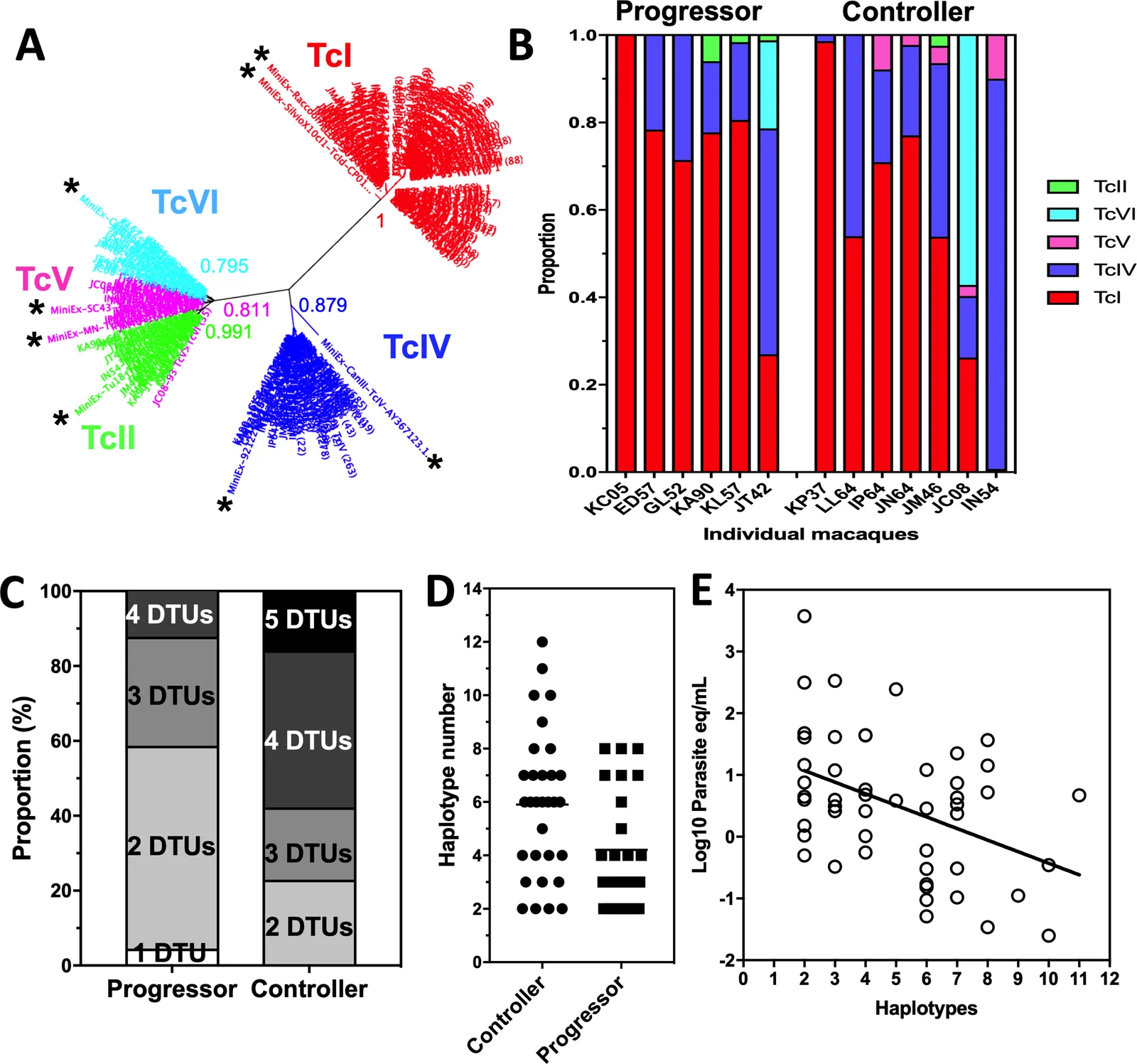
*T. cruzi* parasite diversity in naturally infected macaques. (**A**) Phylogenetic tree of mini-exon sequences from naturally infected macaques, indicating the presence of TcI, TcII, TcIV, TcV, and TcVI parasite DTUs. Parasite DTUs are color-coded as indicated. * indicates sequences from reference strains from each DTU. Bootstrap support of different clades is indicated for each DTU. (**B**) Comparison of parasite DTUs’ respective proportions at the start of follow-up in progressor and controller macaques. Parasite DTUs are color-coded as indicated. (**C**) Comparison of the proportion of infections with different numbers of DTUs in progressor and controller macaques. Most progressor macaques were infected with 1–2 parasite DTUs, while most controller macaques were infected with 4–5 DTUs, χ^2^ = 16.07, *P* = 0.003. (**D**) Comparison of the number of unique mini-exon sequence haplotypes in progressor and controller macaques. Controller macaques presented infections with a significantly larger number of *T. cruzi* haplotypes than progressor macaques, Student’s *t* = 2.48, *P* = 0.016. (**E**) Linear regression between blood parasite burden and the number of infecting *T. cruzi* mini-exon haplotypes. Parasite burden significantly decreased with increasing number of haplotypes, *R* ^2^ = 0.18, *P* = 0.002.

We next genotyped the blood parasites in a subset of 11 macaques using next-generation sequencing of the mini-exon gene, followed by phylogenetic analysis of the sequences. A total of 304 mini-exon sequences were obtained, corresponding to 2–12 unique haplotypes per animal and time point (GenBank accession numbers ON907885–ON908188). Globally, sequences from the cohort clustered with reference sequences belonging to TcI, TcII, TcIV, TcV, and TcVI DTUs (Fig. 3A; Fig. S1), although TcI predominated followed by TcIV, and TcII, TcV, and TcVI were less frequent. As noted before (29), some sequence similarity was observed between mini-exon sequences from these macaques with parasite sequences identified in other mammalian hosts and triatomine vectors from the region, in agreement with these infections being the product of spillover events from local zoonotic transmission cycles. Only one monkey was infected with a single DTU at the start of follow-up (TcI), and all others were infected with mixtures of TcI, TcIV, TcII, TcV, and TcVI parasites in variable proportions, and up to five DTUs could be detected in a single animal (Fig. 3B). Because of the very low prevalence/incidence of infection in these hosts (45), sequential infections with different *T. cruzi* parasite DTUs are extremely unlikely, and the most likely explanation for these multiple infections is simultaneous co-infections with multiple parasite strains. Indeed, triatomine vectors from the region have often been found co-infected with multiple parasite strains/DTUs (26).

Comparison of parasite assemblages between progressor and controller macaques revealed significant differences. Indeed, macaques with a better control of *T. cruzi* parasitemia were predominantly infected with 4–5 parasite DTUs, while those with progressive parasite burden were predominantly infected with only 1–2 parasite DTUs (Fig. 3C, χ^2^ = 16.07, *P* = 0.003). We also assessed the diversity of mini-exon sequence haplotypes detected and accordingly found that macaques with a better control of *T. cruzi* burden presented a significantly higher number of parasite haplotypes compared to those with a progressive parasite burden (Fig. 3D, *t* = 2.48, *P* = 0.016). Furthermore, there was a significant negative correlation between blood parasite burden and haplotype number, with a decreasing burden as the haplotype number increased (Fig. 3E, *R* ^2^ = 0.18, *P* = 0.002). A low sensitivity of our genotyping approach to detect rare haplotypes/DTUs in samples with lower parasite burden could not account for these observations, as this would have resulted in a decrease in haplotypes/DTUs with decreasing parasite burden. Rather, these data suggested that natural infections with a higher diversity of *T. cruzi* strains/DTUs were better controlled by hosts compared to infections with a lower parasite diversity.

**Fig 4:**
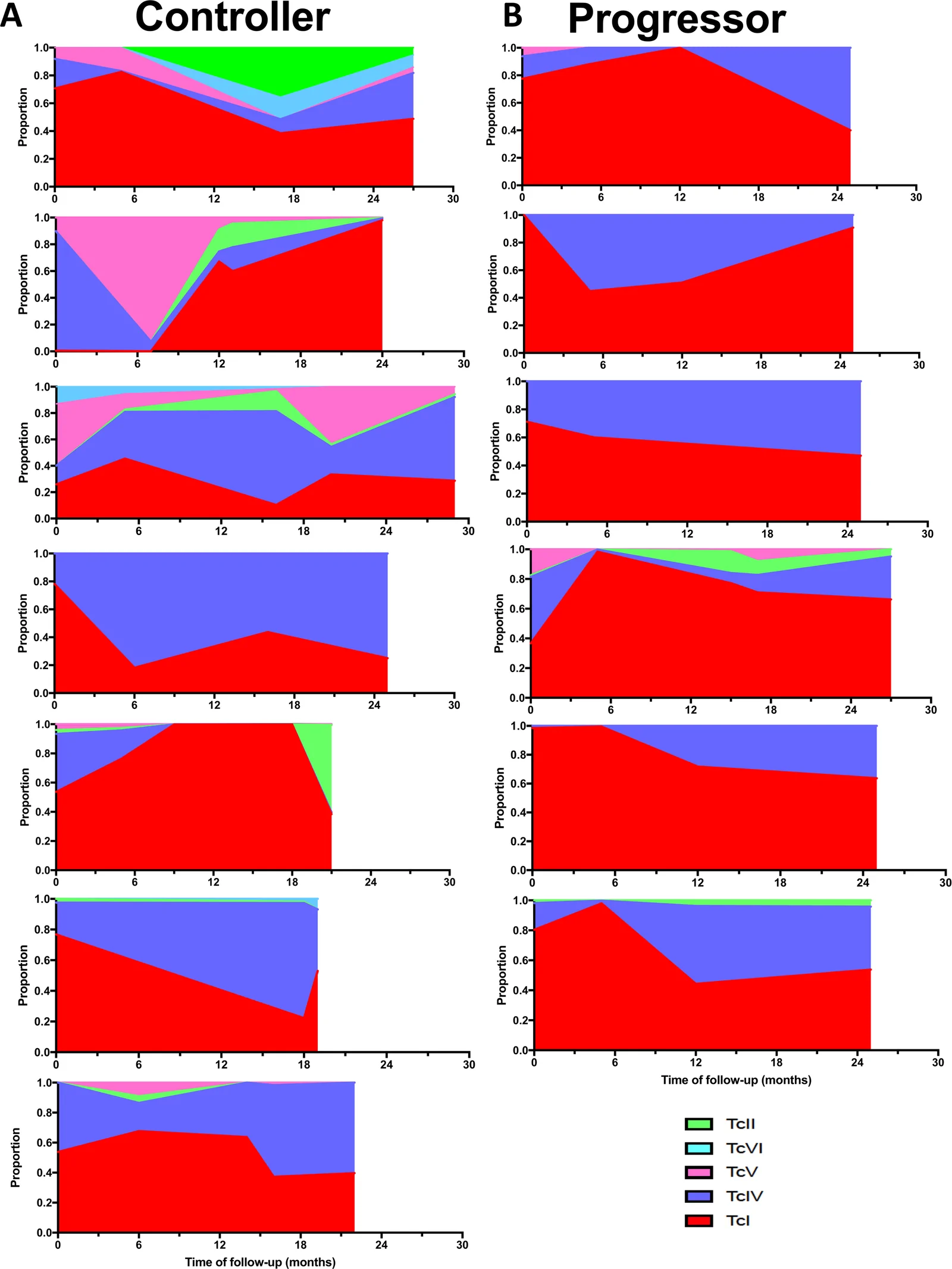
Intra-host *T. cruzi* parasite strain dynamics over time. Changes in *T. cruzi* parasite DTU assemblage were measured over 20–30 months of follow-up and are shown for individual monkeys: (**A**) controllers and (**B**) progressors. Parasite DTUs are color-coded as indicated.

To further evaluate parasite strain interactions and dynamics, we then analyzed potential changes in parasite assemblages over time. Again, two major patterns were observed. In macaques infected with a limited number of parasite DTUs (mostly TcI and TcIV), the relative proportions of each DTU showed limited variation over the 24–30 months of follow-up, with most of these animals corresponding to progressors (Fig. 4A). On the other hand, infections with a higher number of parasite DTUs often resulted in larger changes in the relative proportion of parasite DTUs over time, leading to sequential changes in the predominant DTU, although most, if not all, DTUs persisted over time (Fig. 4B). Thus, different parasite strain dynamics seemed to be occurring in these two groups of macaques, with progressors having rather stable and well-established infections caused by a limited diversity of parasites that were able to persist, while infections with a higher diversity of parasite strains/DTUs led to a limited parasite growth and regular changes in the predominant DTU persisting in these controller hosts.

**Fig 5:**
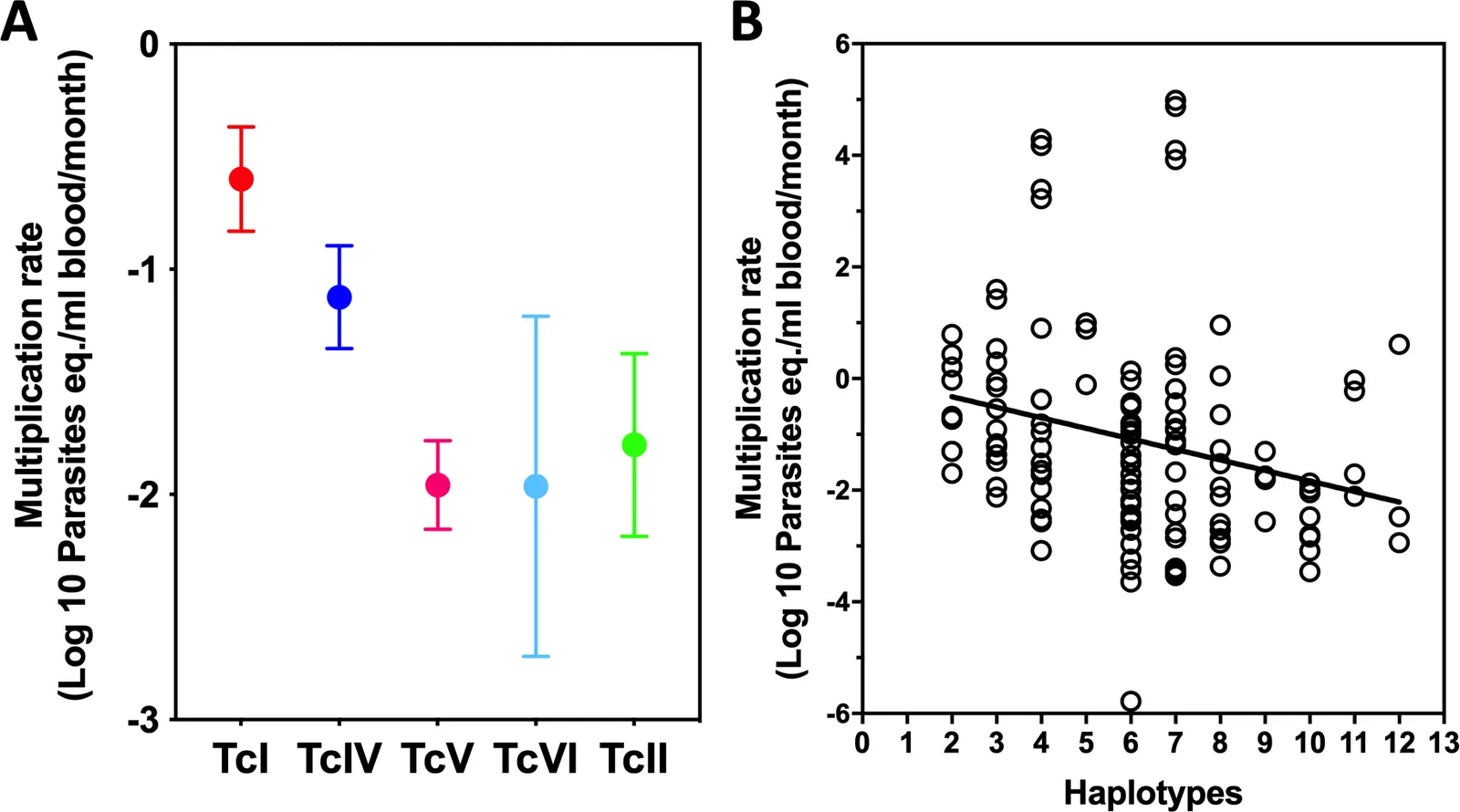
*In vivo T. cruzi* multiplication rate. *T. cruzi* parasite multiplication rate was measured *in vivo* in naturally infected macaques and compared according to parasite DTU (**A**). Multiplication rate varied significantly according to parasite DTU, color-coded as indicated, ANOVA, *F* = 4.0, *P* = 0.004. (**B**) Linear regression of parasite multiplication rate as a function of parasite diversity. Multiplication rate significantly decreased with infections with increasing numbers of parasite haplotypes, *R* ^2^ = 0.067, *P* = 0.003.

These observations suggested that *T. cruzi* strain interactions may be occurring in infections with a higher parasite diversity, resulting in a lower proliferation of the parasites. To assess this point, we calculated DTU-specific parasite multiplication rates in the hosts and found significant differences in the *in vivo* multiplication rate of *T. cruzi* according to the DTU (Fig. 5A, ANOVA, *F* = 4.0, *P* = 0.004). Indeed, TcI parasites presented the highest multiplication rate, followed by TcIV, and TcII, TcV, and TcVI presented the lowest multiplication rates in chronically infected macaques. Such differences may help explain the relative abundance of each DTU in this host population. However, parasite proliferation was also affected by parasite diversity as we detected a significant negative correlation between the multiplication rate and the number of parasite haplotypes present (Fig. 5B, *R* ^2^ = 0.067, *P* = 0.003), with the parasite multiplication rate decreasing when an increasing number of haplotypes were present. These observations strongly supported the hypothesis that increasing parasite diversity in hosts was detrimental to parasite multiplication, possibly due to competition for resources or a stronger immune control through cross-immunity.

**Fig 6:**
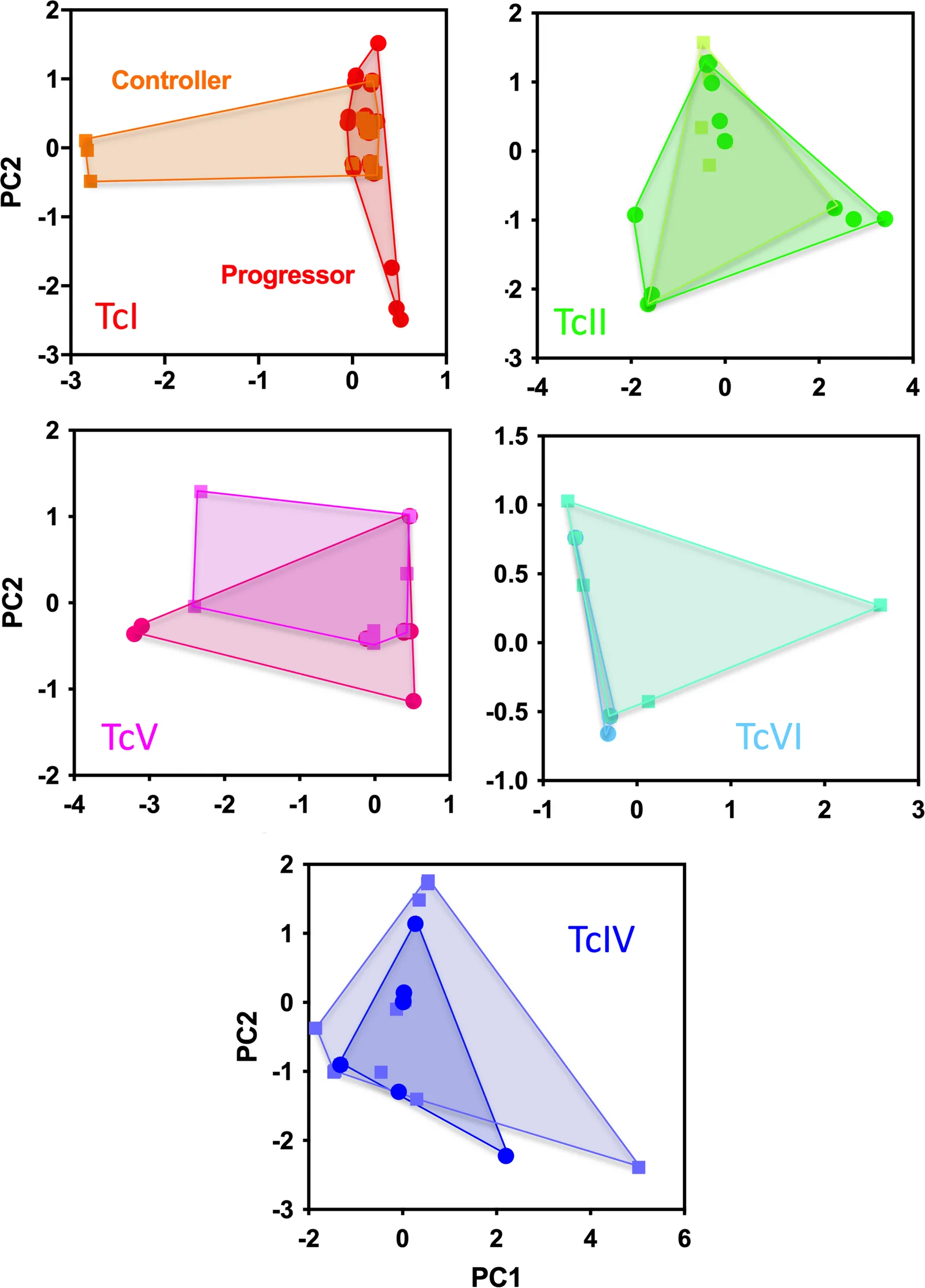
Genetic diversity of *T. cruzi* within DTUs. PCA of mini-exon haplotype sequences was performed for each DTU to compare parasite diversity between progressor and controller macaques. Significant differences in parasite haplotypes between groups were observed for TcI strains (PERMANOVA, *P* = 0.049), but not for TcII, TcIV, TcV, or TcVI parasites (PERMANOVA, *P* > 0.05).

Because parasite strain assemblages seemed to be key for *T. cruzi* control, we also investigated the potential association of specific parasite sequence haplotypes with parasite burden. For this, we performed principal component analysis (PCA) of the mini-exon sequences from each DTU among progressor and controller macaques (Fig. 6). We detected significant differences in sequence haplotypes between progressor and controller macaques only for TcI parasites (Fig. 6A, PERMANOVA, *P* = 0.049), but not for any of the other DTUs (Fig. 6B through E, *P* > 0.05). Thus, genetic differences among strains within each DTU may play a lesser role than DTU composition in determining parasite burden and disease progression. However, it should be noted that *T. cruzi* genetic diversity within DTUs may have been limited in this cohort of hosts from the same habitat.

### Electrocardiographic assessment of cardiac function

**Fig 7:**
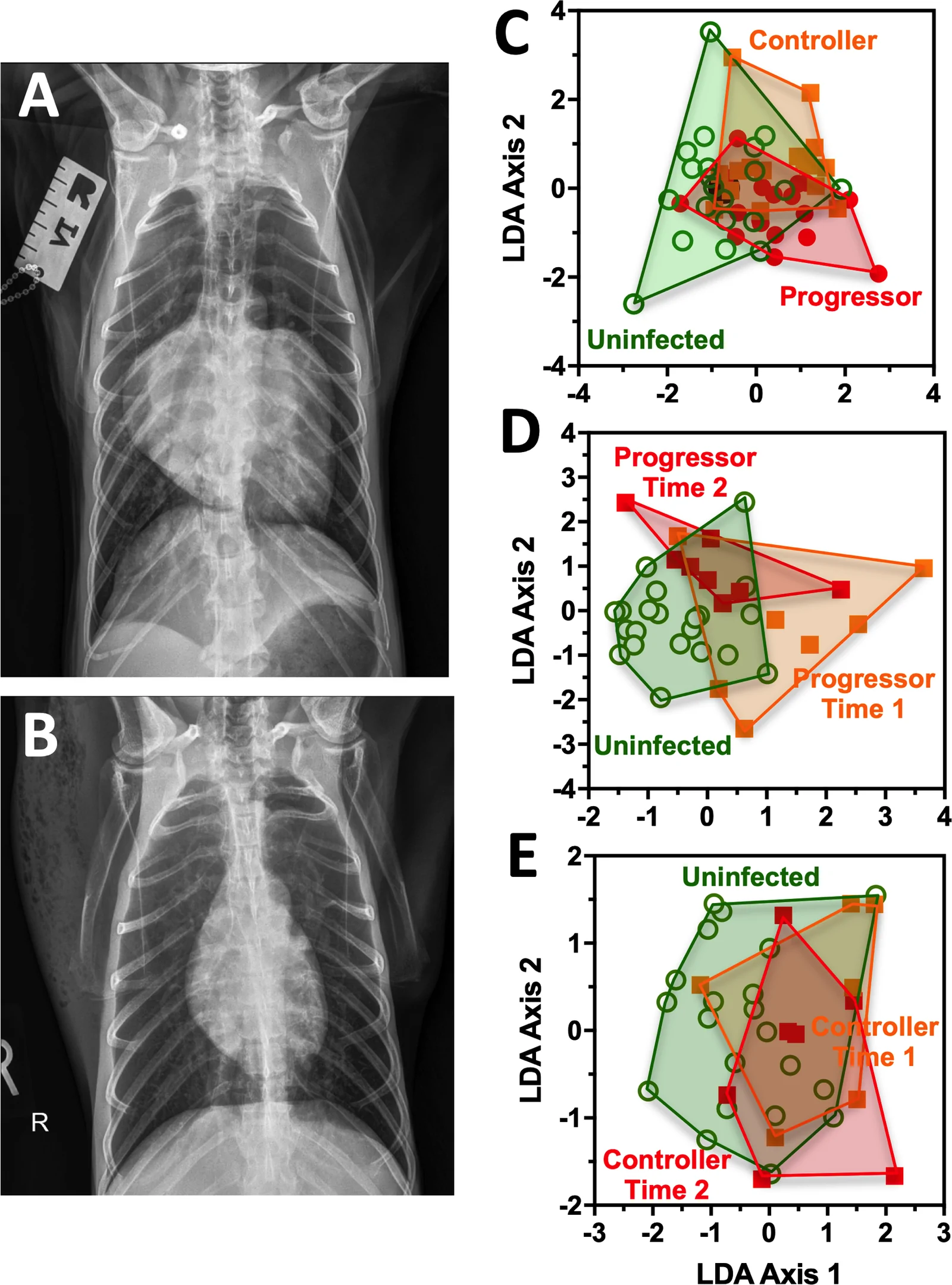
Cardiomegaly and ECG alterations in Chagasic macaques. Thoracic X-ray image of a Chagasic (**A**) and uninfected age-matched macaque (**B**) showing pronounced cardiomegaly in the Chagasic animal. (**C**) LDA of ECG parameters of uninfected, and progressor and controller macaques, indicating significant differences in their ECG profiles, PERMANOVA, *P* = 0.036. Up to 63.6% of animals could be correctly classified into their respective groups by the LDA. (**D**) LDA of ECG parameters of progressor macaques at two time points about 6–15 months apart, indicating significant changes in ECG profiles over time, PERMANOVA, *P* = 0.015. Up to 73.0% of animals could be correctly classified into their respective groups by the LDA. (**E**) LDA analysis of ECG parameters of controller macaques at two time points about 6–15 months apart, indicating no significant changes in ECG profiles over time, PERMANOVA, *P* = 0.23, although up to 64.7% of animals could be correctly classified in their respective groups by the LDA.

Next, we also evaluated cardiac function in this cohort of Chagasic macaques, as well as in age- and sex-matched uninfected control animals, through electrocardiographic recordings. Measurement of *P* wave, PR, QT, QRS, and RR intervals indicated that there were no significant differences in individual ECG parameters between Chagasic and uninfected macaques. However, integrated analysis of these parameters through linear discriminant analysis (LDA) showed that ECGs from Chagasic macaques differed from those from uninfected controls (PERMANOVA, *P* = 0.007), and 75.6% of individuals could be correctly reclassified between Chagasic and controls based on their ECG profiles (Table S2). These data indicated that while major arrhythmias were not detected, Chagasic macaques presented small conduction defects suggestive of the onset of cardiac alterations. Interestingly, one Chagasic macaque presented major cardiomegaly after only one year of infection, associated with severe kyphosis and generalized muscle atrophy, suggestive of a rapid and advanced clinical disease in this animal (Fig. 7A and B).

Further analysis of ECG parameters based on the blood parasitemia of Chagasic macaques (progressor vs controller) also revealed significant differences in ECG profiles between these two groups, which differed from uninfected controls (Fig. 7C, PERMANOVA, *P* = 0.036), and 63.6% of animals could be correctly reclassified by the LDA. A subset of animals had two ECG recordings available taken 8–15 months apart during our follow-up, allowing us to examine potential changes over time. Again, no significant changes were detected among single ECG parameters. However, the LDA indicated that ECGs from macaques classified as progressors based on parasitemia presented significant differences over time and with uninfected controls (Fig. 7D, PERMANOVA, *P* = 0.015), while ECGs from controller macaques remained unchanged over time (Fig. 7E, PERMANOVA, *P* = 0.23). Together, these observations indicate that macaques with increasing parasitemia present more rapidly progressing ECG alterations, while those with more controlled parasitemia have no or more limited progression of the ECG alterations over the follow-up period.

### Biomarker of disease progression

**Fig 8:**
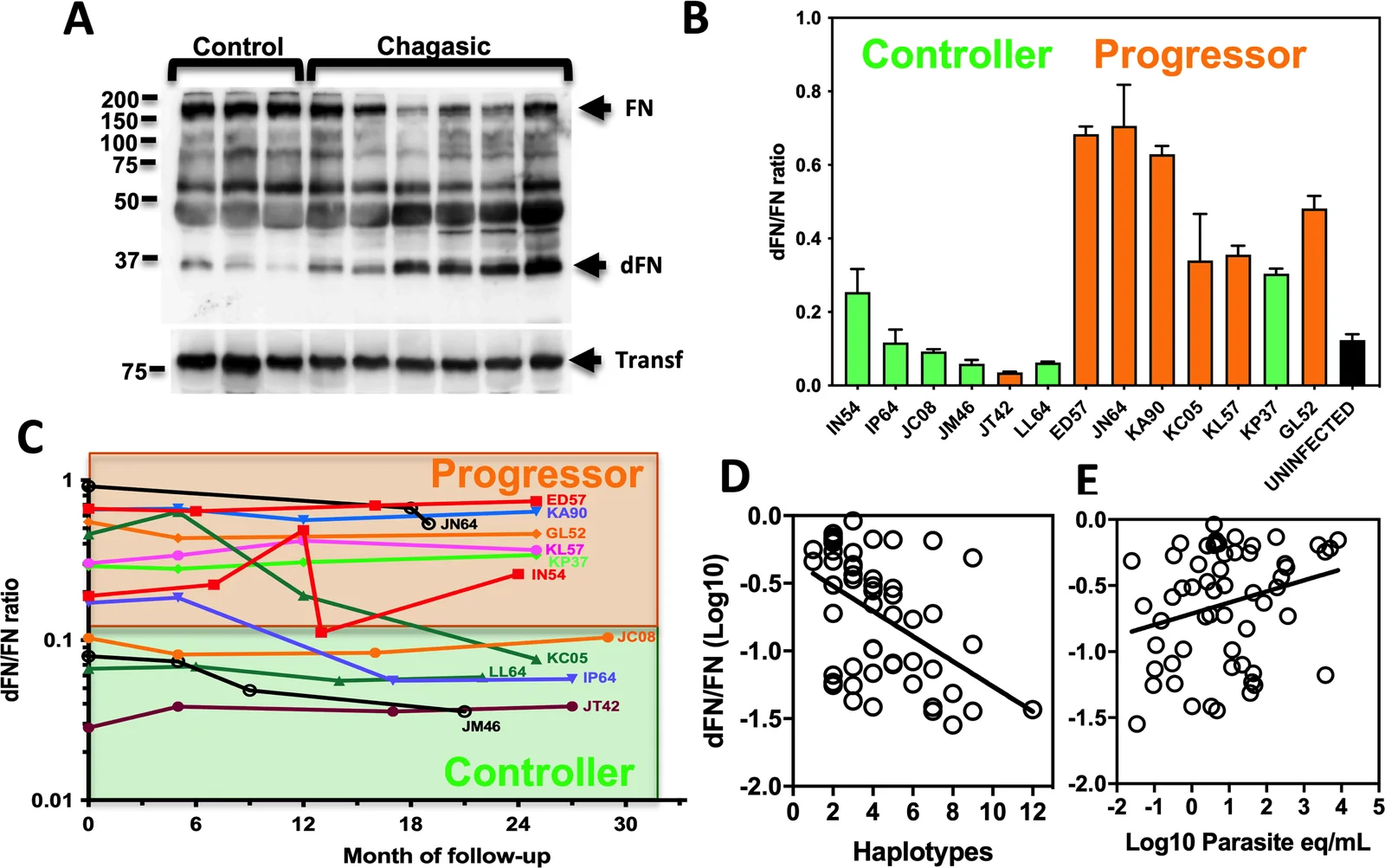
Plasma fibronectin degradation as a biomarker of disease progression. Plasma fibronectin degradation was measured by western blot (**A**). High-molecular-weight fibronectin (FN) is partially degraded in Chagasic macaques compared to uninfected controls, which results in an increase in lower-molecular-weight fragments, including a fragment of about 37 kDA (dFN). Plasma transferrin (Transf) was used as the loading control. Densitometric quantification of fibronectin degradation (**B**) indicated levels comparable to uninfected macaques in controller Chagasic macaques and significantly increased levels in progressor Chagasic macaques (*t* = 4.5, *P* < 0.0001). (**C**) Measurement of fibronectin degradation over time indicated very stable levels in both controller and progressor macaques over 20–30 months of follow-up. Color-coded lines and symbols indicate individual macaques, whose ID is indicated. (**D**) Fibronectin degradation was negatively correlated with parasite haplotype number, *R* ^2^ = 0.23, *F* = 15.64, *P* = 0.0002, and (**E**) positively correlated with blood parasite burden, *R* ^2^ = 0.07, *F* = 4.03, *P* = 0.049).

To expand on our assessment of disease progression in these macaques, we then measured plasma levels of fibronectin, as this protein has been proposed as a biomarker for Chagas disease. Indeed, the levels of degraded fibronectin have been found to be increased in Chagasic patients as well as in mouse models (11 – 13), as it is a proposed substrate for cruzipain, a parasite cysteine protease (48). Western blot analysis of macaque plasma proteins indicated that Chagasic macaques indeed presented a reduced amount of high-molecular-weight intact fibronectin, together with a higher amount of multiple degradation fragments of lower molecular weight, compared with uninfected animals (Fig. 8A). In particular, a 37-kDa degradation fragment was observed, which may correspond to a comparable low-molecular-weight fragment identified in Chagasic patients and experimentally infected mice (11 – 13). Further quantitative analysis of fibronectin degradation and its changes over time indicated that individual levels were rather stable over the 24–30 months of follow-up and that macaques controlling their blood parasite burden had a plasma fibronectin level similar to that of uninfected animals, while those with progressive parasite burden presented a significantly elevated fibronectin degradation (Fig. 8B and C, *t* = 4.5, *P* < 0.0001). Finally, fibronectin degradation was significantly correlated with parasite haplotype number, and infections with a lower number of haplotypes were associated with a higher level of fibronectin degradation (Fig. 8D, *R* ^2^ = 0.23, *F* = 15.64, *P* = 0.0002). Fibronectin degradation also significantly increased with increasing blood parasite burden (Fig. 8E, *R* ^2^ = 0.07, *F* = 4.03, *P* = 0.049). These observations suggested that fibronectin degradation may be associated with disease severity and may be a valuable prognostic biomarker of disease progression.

### Integrative modeling of disease progression

Finally, we built a statistical model to identify key variables allowing us to predict Chagas disease progression in our cohort of naturally infected macaques, based on the progression in blood parasite burden (progressors vs controllers). The best model provided a rather good fit of the data (*R* ^2^ = 0.55, *P* = 0.004) and included the duration of the infection, fibronectin degradation level, QRS duration, and parasite haplotype number as variables (Table 1). Another model provided a somewhat better fit (*R* ^2^ = 0.63, *P* = 0.004), although AIC indicated that it was slightly worse than the best model (AIC = 28.7 vs 26.6), and this model included the same variables plus the body weight change at the time of infection (Table 1). These models strongly suggested that changes in blood parasite burden over time were associated with the duration of infection, parasite genetic diversity, and changes in ECG profiles, and that fibronectin degradation may serve as a valuable biomarker of disease progression. Body weight loss at the time of infection, which may reflect the severity of the acute phase, may also be an important predictor of disease development at later stages.

**TABLE 1:** Logistic models for blood parasitemia changes over time^*a*^

Model #1	X^2^	*P*	*R* ^2^	AIC
	15.22	0.0043*	0.55	26.6

Term	Parameter estimates		Effect likelihood ratio tests	
	Estimate	Std Error	*X* ^*2*^	*P*
Intercept	−0.949	4.059		
Duration of infection	−0.112	0.064	9.361	0.0022*
Fibronectin degradation	−0.356	0.260	2.756	0.0969
QRS duration	211.384	136.695	4.225	0.0398*
Haplotype number	0.898	0.503	5.418	0.0199*

Model #2	X^2^	*P*	*R* ^2^	AIC
	17.23	0.0041*	0.63	28.7

Term	Parameter estimates		Effect likelihood ratio tests	
	Estimate	Std Error	X^2^	*P*
Intercept	−2.210	5.368		
Duration of infection	−0.145	0.092	10.080	0.0015*
Fibronectin degradation	−0.477	0.373	3.042	0.0811
QRS duration	289.089	172.827	5.967	0.0146*
Haplotype number	1.156	0.636	6.604	0.0102*
Body weight change	1.333	1.127	2.014	0.1558

^*a*^ * indicates statistically significant *P* values (<0.05).

## DISCUSSION

Chagas disease progression during the chronic phase remains poorly understood, and naturally infected macaques offer an unprecedented opportunity to investigate this process. Thus, we evaluated for the first time intra-host *T. cruzi* strain dynamics in association with parasitological and clinical indicators of disease progression in rhesus macaques chronically infected for 1–6 years.

Blood parasite burden is a key parasitological indicator, and it is the primary outcome to assess disease progression and response to treatment in clinical trials of drug therapies against Chagas disease (7, 49 – 51). We found that at the population level, blood parasite burden was low but rather stable over time in our untreated macaques, which is similar to previous observations from placebo-treated human patients in the chronic phase. However, we also detected important individual variations in changes in parasitemia over time, suggestive of differences in parasite control, with some animals presenting a gradual increase in parasitemia while others presented a decrease in parasitemia over time. We then assessed possible explanations for these differences in parasitological profiles between the two groups.

We found that differences in blood parasite burden profiles were in large part associated with parasite genetic diversity, more specifically with the parasite strain assemblage infecting each host, as well as associated with more rapid changes in ECG alterations. Indeed, we first confirmed the circulation of a large diversity of DTU, expanding previous work (29). While the use of the mini-exon as a single marker for genotyping *T. cruzi* has limitations, particularly the inability to assess the exact number of parasite strains within DTUs (31, 52, 53), it allowed a reliable follow-up of parasite dynamics over time. Future studies based on additional markers or the isolation of parasites may provide more detailed data on infecting strains, but have limitations in terms of sensitivity and can be biased toward strains growing better *in vitro* (31, 54). Thus, our data shed some new light on *T. cruzi* strain interactions in such multiple infections. Indeed, a major finding of our study was that infections with an increased diversity of parasite strains/DTUs were associated with a decreasing parasitemia and a lower *in vivo* multiplication rate of the parasite compared to infections with a more limited parasite diversity. Accordingly, these observations suggest that in macaques, infections with mixtures of *T. cruzi* strains/DTUs do not benefit the parasite by allowing a severe “super-infection” to develop, but rather favor the host by allowing for a better control of the parasite. This reduced parasite proliferation may be due to multiple non-exclusive mechanisms (35, 55). For example, it may involve (1) direct interference, in which strains produce substances to inhibit each other; (ii) resource competition, such as for nutrients or host infection sites (tissue/cell specificity of strains) that are used and no longer available for other strains and limit their growth, which is an indirect form of competition; or (iii) a stronger immune response that controls parasite infection through cross-reactivity among strains. In this case, strains with higher similarity suffer from increased immune pressure, and more divergent strains may be favored by a reduced antigenic overlap allowing for some immune evasion (35, 38, 56). In this respect, it is worth noting that, in spite of the detrimental interactions involving multiple strains, parasite diversity is largely maintained over time in these chronically infected macaques, although at low levels of parasitemia. Also, as cardiac disease progression appears slower when a high parasite diversity is present, it is likely that inflammatory responses leading to myocarditis are decreased. Thus, our overall observations provide the first evidence that *T. cruzi* proliferation and cardiac disease progression may be limited as the diversity of infecting strains/DTUs increases, and further studies should help disentangle how these possible mechanisms may be involved. Nonetheless, it should be noted that a limitation of our study is that we could only assess parasite diversity in the blood, and different patterns of strain/DTU diversity and their interactions may occur in other tissues.

Previous studies of experimental infections in mice with a mixture of *T. cruzi* strains have mostly focused on the comparison of combinations of pairs of strains vs each strain alone (40 – 42), and to our knowledge, mixtures of more than two strains have not been evaluated in experimental infection models. The experimental co-infection with two strains of *Trypanosoma brucei* in mice similarly resulted in the suppression of individual strains and increased host survival, indicative of strain competition in the host (39). Natural infections with *Plasmodium vivax* are composed of mixtures of closely related parasites, and mutation and selection processes could be identified over time in patients, indicative of parasite evolution in the host (57).

We also detected differences in *in vivo* multiplication rates among *T. cruzi* DTUs. Multiplication rate is an important determinant of the virulence and fitness of pathogens in hosts (58), and the observed differences among DTUs may help explain, at least in part, the relative proportions/abundance of the respective *T. cruzi* DTUs in macaques. Other host species from the region, such as cats, dogs, or rodents (32, 33, 46), seem to harbor somewhat different proportions of *T. cruzi* DTUs, and further studies of strain dynamics in these hosts should help better characterize parasite traits, which may vary according to host species.

We also observed a limited role of specific parasite genotype/haplotype on disease progression compared to the effect of strain mixtures. This appears contrary to many observations of individual strain biological variability *in vitro* and *in vivo* (16 – 20). However, within DTUs, parasite diversity was overall low in our rather homogenous cohort of parasites and hosts from the same habitat. Comparison with a broader diversity of strains from other geographic regions may be needed to assess this point as the geographic differentiation of *T. cruzi* strains is also likely contributing to biological differences (52, 59).

Interestingly, our data on body weight loss at the time of *T. cruzi* infection, although rather crude, also suggest that this may be a very early indicator of the long-term outcome of infections during the chronic phase. However, as the acute phase is rarely identified in patients, this may be difficult to confirm. On the other hand, plasma fibronectin degradation is emerging as a valuable biomarker of disease progression as proposed before (11 – 14). Indeed, we found that it was specifically increased in macaques with increasing parasite burden and progressive alterations in ECG recordings. The development of novel assays for an easier and more quantitative measurement of fibronectin degradation fragments than western blot would thus be helpful for its further evaluation. Also, measurements of cruzipain protease levels may provide important information as it may mediate fibronectin degradation (48).

**Fig 9:**
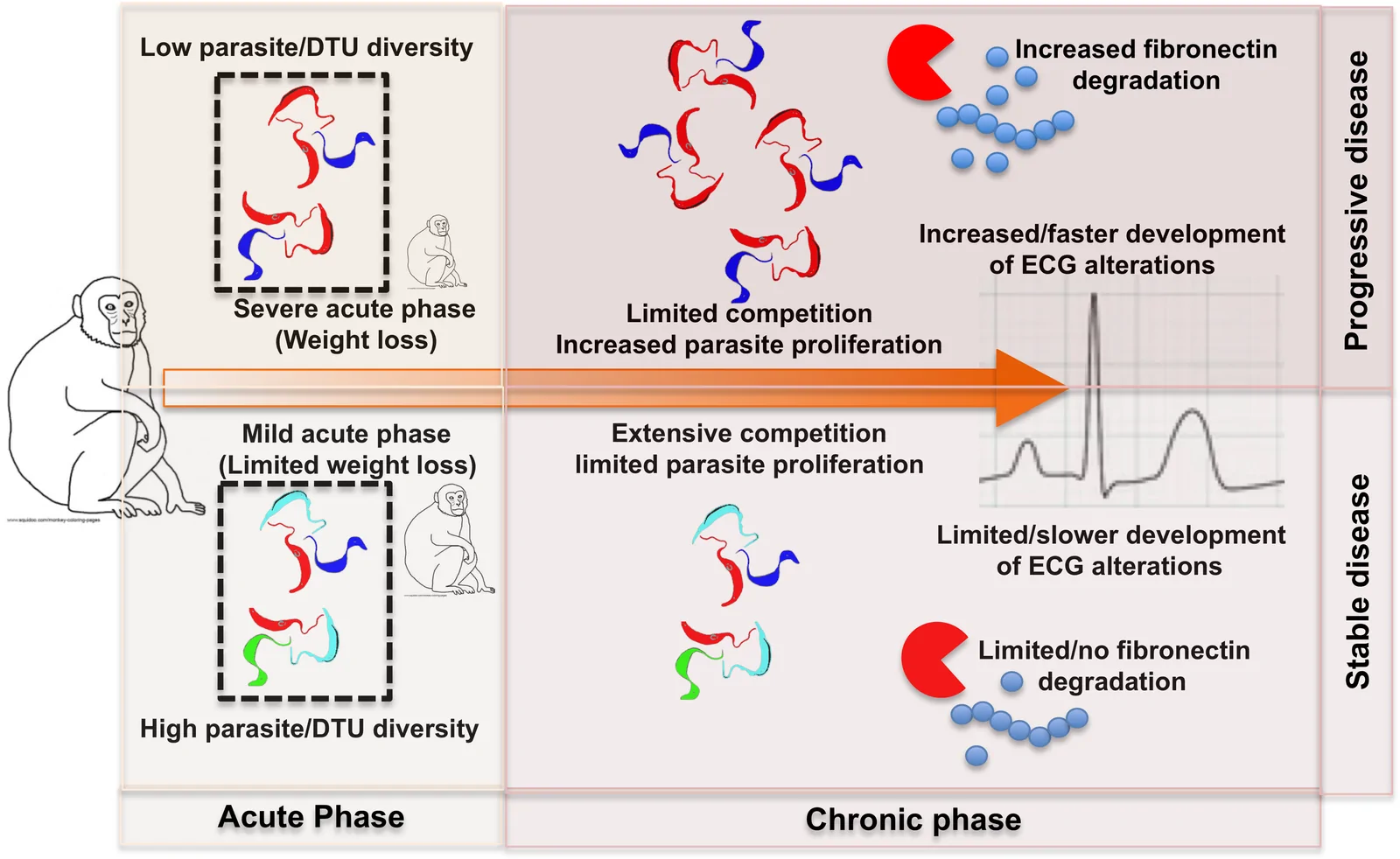
A framework for disease progression in Chagasic macaques. We propose that disease progression in the chronic phase is in large part determined by the onset of infection according to *T. cruzi* parasite diversity. See the text for additional explanations.

Finally, our study provides a general framework for a better understanding of the relationship between parasitological and clinical disease progression in naturally infected Chagasic macaques. Indeed, our data suggest that disease progression in the chronic phase is in large part determined by the onset of infection according to parasite diversity (Fig. 9). Thus, on the one hand, infection with a limited diversity of parasite strains/DTUs would result in a more severe acute phase and a favorable environment for their multiplication and long-term persistence in the host at increasing levels. This would be associated with more rapidly progressing ECG alterations, which may eventually lead to severe arrhythmias and cardiac dysfunction. Also, increased levels of plasma fibronectin may serve as a biomarker of this situation. On the other hand, infection with a higher diversity of parasite strains/DTUs would result in a milder acute phase, with limited parasite proliferation, and lead to lower levels of persisting parasites. This would be associated with no or very slow development of ECG alterations, which may not progress toward severe cardiac dysfunction. Also, normal levels of plasma fibronectin may serve as a biomarker of this outcome. While additional factors, such as host intrinsic factors (60, 61), are likely to also affect disease progression, our simple framework allows for further evaluating the mechanisms of pathogenesis in Chagasic hosts.

Indeed, this framework may help explain differences in clinical profiles and disease progression in Chagasic patients, and warrants further studies aimed at better assessing *T. cruzi* parasite diversity in patient cohorts associated with their clinical profile (31, 34). For example, a recently reported acute case from Belize was found to be co-infected with a mixture of TcIV, TcII, and TcV parasites and effectively treated with nifurtimox (62), and treatment effectiveness may have benefited from the multiplicity of parasite strains interacting. Also, preclinical models of *T. cruzi* experimental infection rely mostly, if not exclusively, on infections with single parasite strains, which accordingly would be the most stringent models for testing the efficacy of drugs or vaccines.

In conclusion, our integrative evaluation of parasitological and clinical aspects of *T. cruzi* infection in naturally infected rhesus macaques, with details of the intra-host parasite DTU dynamics over prolonged time, reveals unique interconnections between parasite genetic diversity and disease progression. In particular, we provide evidence of the multiplicity of infection in the host as a major driver of disease progression. It would be of key interest to further assess the potential mechanisms underlying the interactions among multiple strains, and such empirical data in natural infections are critical to complement other more theoretical approaches, given the paucity of experimental data. Fibronectin degradation is also emerging as a valuable biomarker in Chagas disease, which may eventually lead to a better prognosis for patients. Finally, further testing and refining the proposed framework for Chagas disease progression in Chagasic patients is warranted to reach a better understanding of pathogenesis and ultimately improve patient care and treatment.

## MATERIALS AND METHODS

### Animals

Rhesus macaques (*Macaca mulatta*) of Indian and Chinese ancestry were housed in the breeding colony of the Tulane National Primate Research Center (TNPRC) in species-appropriate social groups in outdoor enclosures in accordance with the Animal Welfare Act, the Guide for the Care and Use of Laboratory Animals, and other federal statutes and regulations. Both Indian-origin and Chinese-origin macaques are used in research, and they differ in appearance and genetic background. Differences in susceptibility to infections have been described between the subspecies for some pathogens (63), but no information is available regarding susceptibility to *T. cruzi* infection. Outdoor enclosures are constructed of wire mesh walls and a roof with natural ground cover and a variety of perches, shelters, and swings. Water is available *ad libitum*, and a standard, commercially formulated nonhuman primate diet is provided daily and supplemented with fresh fruit, vegetables, and/or other forage materials several times a week. In addition to daily observations, all macaques receive routine examinations and preventative medicine treatments (vaccines, anthelmintics) during semiannual health assessments. Blood samples are also collected for colony surveillance and diagnostic purposes. All breeding and management protocols are reviewed and approved by the Tulane University Institutional Animal Care and Use Committee. Animals were identified as seropositive for *T. cruzi* parasites (Table S1) during routine colony surveillance. Infections occurred naturally upon contact with infected *Triatoma sanguisuga*, the main vector in Louisiana (26), which may occasionally be present in outdoor enclosures. Additional blood samples were collected in subsequent routine examinations to allow for *T. cruzi* parasite monitoring, but unfortunately, no historic samples from when animals seroconverted were available to assess initial parasite burden or genotypes. Between two and five blood samples per animal were thus collected over a 2.5-year period of follow-up, although for a few animals, only one sample was available. Because sampling only occurred during routine care, it is biased for females as these were followed more closely than males in the breeding colony (Table S1). When possible, electrocardiographic recordings were performed to assess cardiac function, yielding between one and four ECG recordings per animal over the study period. Age- and sex-matched, healthy, uninfected animals were used to obtain control blood samples and ECG recordings for comparison. All procedures were performed on sedated animals. The study protocol was approved by the Tulane Institutional Animal Care and Use Committee.

### *Trypanosoma cruzi* infection diagnostic

*Trypanosoma cruzi* infection was initially diagnosed through a Multiplexed Fluorometric Immunoassay (MFIA, Charles River Laboratories), which simultaneously tests for multiple pathogens and is routinely performed as part of health monitoring of the TNPRC breeding colony. Infection was then confirmed using Stat-Pak rapid immunochromatographic test (Chembio, Medford, NY), two diagnostic PCR reactions targeting nuclear satellite DNA (Sat. DNA PCR, TcZ1/TcZ2 primers) (64), and parasite kinetoplast DNA (kDNA PCR, Tc121/Tc122 primers) (65). Body weight, medical histories, and X-ray images were obtained from health records from routine care of the animals. Changes in body weight at the time of infection were obtained by calculating the difference in weight at the time of seroconversion and the previous measurement.

### Blood parasite burden

Blood parasite burden was measured using a validated and standardized duplex qPCR method based on Taq-Man probes, targeting *T. cruzi* SatDNA and RNAse P gene as an internal amplification control (50). Because blood parasite burden in chronically infected hosts may be close to or even below the detection limit (66, 67), targeting a multi-copy sequence allows increased sensitivity. This assay is used in clinical trials evaluating treatment effectiveness in Chagasic patients and has been extensively validated (68). While some possible differences in sensitivity among parasite DTUs have been noted (for TcId and TcIe, not detected in these macaques, and for TcIV, but not for the other DTUs), the limited number of parasite strains tested does not allow for any generalization or correction/adjustment of the measured parasite burden (68). Briefly, DNA was extracted from 200 µL of blood with the Qiagen DNEasy Blood and Tissue Kit as per the manufacturer’s instructions, and amplifications were performed with 5 µL of DNA in a final volume of 20 µL. Uracil-DNA Glycosylase (Thermo Fisher Scientific) was added to the reaction mix, as a carry-over contamination control, and TaqMan RNase P Control Reagents Kit (Applied Biosystems) was used. Cycling conditions will be as follows: a first step of 2 min at 50°C and a second step of 10 min at 95°C, followed by 40 cycles at 95°C for 15 s and 58°C for 1 min. All samples were analyzed in duplicate. The qPCR results were converted to parasitic load (parasite equivalents/mL of blood) using a standard calibration curve, and data were log-transformed for statistical analysis.

### *T. cruzi* parasite genotyping

Parasite genotyping was performed using a multiplex PCR targeting the mini-exon sequence, which gives PCR products of different sizes according to the DTU (69), and TrypME3 and TcCH primers, which amplify a larger fragment of 500 bp of this marker from all DTUs (53), as in previous studies (32). Briefly, amplicons were pooled for each sample and sequenced on a MiSeq (Illumina) platform. Reads were competitively mapped to mini-exon reference sequences from each DTU (70), and sequence variants were identified with the FreeBayes SNP/variant tool (71). Phylogenetic trees based on maximum likelihood were built using FastTree (72) to assess haplotype clustering with reference sequences from all DTUs. The number of reads of each unique haplotype and for parasite DTUs was used to calculate their relative proportions, and only those representing at least 1% of the reads were included in the analysis. Mini-exon sequences were deposited in the GenBank database under accession numbers ON907885–ON908188.

Absolute parasite burden for each DTU was calculated by multiplying their relative proportions with the corresponding total parasite burden of each sample measured by qPCR at each time point. From these data, DTU-specific parasite multiplication rates were measured by calculating absolute changes in parasite number from each DTU between the time points of our follow-up and expressed as parasite equivalents/mL of blood/month. Data were further log-transformed for statistical analysis.

Mini-exon sequence haplotype diversity was further assessed by principal component analysis. Because of the large sequence difference among DTUs, separate analyses were performed with sequences from each DTU. The statistical significance of differences among groups of sequences was analyzed by permutation ANOVA (PERMANOVA) based on 10,000 permutations and Jukes-Cantor distances among sequences. Analyses were performed in PAST 4.0 (73).

### Electrocardiographic recording

Six lead electrocardiographic recordings were performed on sedated macaques to assess cardiac function using a DRE TrueVET ECG-1 Single Channel recorder at a speed of 25 mm/s and 40 mm/mV. Most animals were assessed once, but a subset had repeated ECG recordings at 8- to 12-month intervals to assess potential changes over time. For all recordings, the duration of the P wave, PR, RR, QRS, QT, and QTc intervals was measured (an average of 10 beats for each animal), and individual parameters were compared between groups using the Student’s *t*-test. For multivariate analysis, ECG parameters were integrated into the LDA (74), and groups were compared according to the first and second LDA axes. One-way PERMANOVA was used to assess the statistical significance of differences among groups with 10,000 permutations based on Manhattan distances. The confusion matrix of the LDA was also used to evaluate the accuracy of the reclassification of individual macaques among groups based on the similarities/differences in their ECG patterns.

### Measurement of plasma fibronectin

Fibronectin and its degradation fragments were identified in plasma samples by western blot with a commercial antibody (Rabbit anti-fibronectin; Sigma-Aldrich) and quantified by densitometric analysis as before (14). Plasma samples were stored with protease inhibitors (protease inhibitor cocktail: 10 mM Iodoacetamide; 1 mM N-Methylmaleimide; 1 mM Benzamidine; 1 mM EDTA; 1 mM Sodium Orthovanadate; 1 mM PMSF; 50 mM Tris Base [all from Sigma]), and 20 µg of proteins in Laemmli buffer was separated on a 12% polyacrylamide gel and transferred onto nitrocellulose membranes. Fibronectin and its degradation products were detected with a 1:800 dilution of polyclonal antibody (Rabbit anti-fibronectin; Sigma-Aldrich) followed by a peroxidase-labeled secondary antibody [Goat anti-Rabbit IgG (H + L) HRP Antibody, EMD Millipore Corp.] at a 1:10,000 dilution. Plasma transferrin was used as the sample loading control after detecting fibronectin. The nitrocellulose membranes were stripped in a stripping solution of 100 mM 2-mercaptoethanol, 2% (wt/vol) SDS, 62.5 mM Tris-HCl, pH 6.7, at 50°C for 30 min and reprobed with an anti-transferrin rabbit polyclonal antibody (Proteintech) at a dilution of 1:1,000, followed by the secondary antibody [Goat anti-Rabbit IgG (H + L) HRP Antibody, EMD Millipore Corp., 1:10,000 dilution]. Blots were scanned on a Bio-Rad ChemiDoc Imaging System for densitometric analysis, and image analysis was performed using ImageJ. Fibronectin levels were normalized to transferrin and expressed as the ratio of degradation fragments/intact fibronectin (in arbitrary units). Data were log-transformed for statistical analysis.

### Statistical modeling

We used logistic regression to model profiles of blood parasite burden as a function of parasite diversity, duration of infection, ECG alterations, and other clinical variables. Several models were built based on different sets of variables, and we used Akaike information criteria for model comparison and selection of the best model(s). Analyses were performed in SAS JMP 9.0.

## Data Availability

DNA sequence data that support the findings of this study have been deposited in GenBank with the accession numbers ON907885–ON908188. Additional data are available within the paper and its supplementary information files or from the corresponding author upon reasonable request.

## SUPPLEMENTAL MATERIAL

Supplemental material spectrum.04236-22-s0001.pdf Supplemental figure and tables.

ASM does not own the copyrights to Supplemental Material that may be linked to, or accessed through, an article. The authors have granted ASM a non-exclusive, world-wide license to publish the Supplemental Material files. Please contact the corresponding author directly for reuse.
