# Supplementary material for: Intra-host Trypanosoma cruzi strain dynamics shape disease progression: the missing link in Chagas disease pathogenesis
Source: Microbiol Spectr. 2023 Sep 5;11(5):e04236-22. doi: 10.1128/spectrum.04236-22 (PMC10581044; doi:10.1128/spectrum.04236-22)
Supplement: Supplemental material — Supplemental figure and tables. [file spectrum.04236-22-s0001.pdf]

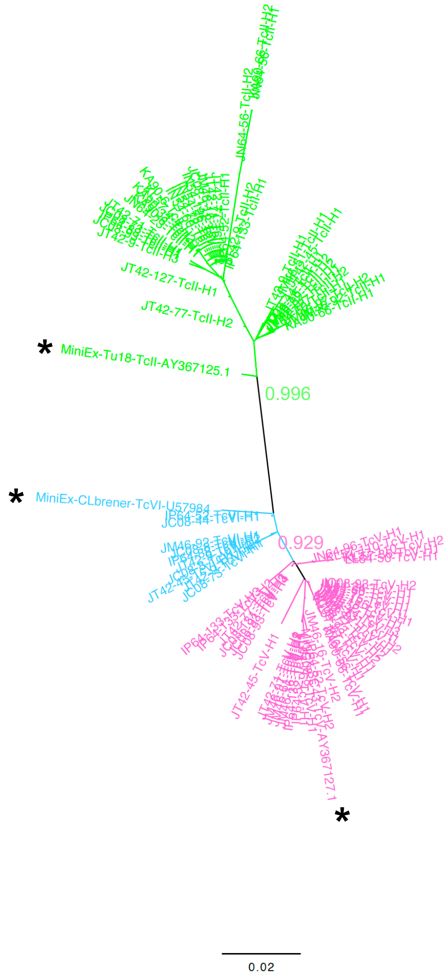

Supplemental Figure 1. Phylogenetic analysis of TcII, TcV and TcVI sequences from Chagasic macaques.

Sequences from the corresponding DTUS from Figure 3A were selected for the analysis. * indicates sequences from reference strains (Tu18 for TcII, CLBrener for TcVI and SC43 for TcV). Bootstrap support for the main clades in indicated.

Supplement Table 1. Demography of Chagasic macaques

ID	Sex	Species	Ancestry	Age (Years)	Time Infected (Years)
**DI83**	M	*M.mulatta*	Chinese	19	1
**ED57**	F	*M.mulatta*	Chinese	18	5
**EP36**	F	*M.mulatta*	Indian	17	4
**GI52**	F	*M.mulatta*	Chinese	15	3
**GL17**	F	*M.mulatta*	Indian	15	6
**HA67**	F	*M.mulatta*	Indian	14	4
**HA87**	F	*M.mulatta*	Indian	14	6
**HF22**	F	*M.mulatta*	Indian	14	4
**HN51**	M	M.mulatta	Indian	13	6
**HN75**	F	*M.mulatta*	Indian	13	6
**HV76**	F	M.mulatta	Indian	13	5
**IN54**	F	*M.mulatta*	Indian	11	4
**IP64**	F	*M.mulatta*	Chinese	11	6
**JC08**	F	*M.mulatta*	Indian	11	4
**JL71**	M	*M.mulatta*	Indian	10	6
**JM46**	M	*M.mulatta*	Indian	10	3
**JN64**	F	M.mulatta	Indian	10	2
**JN71**	F	*M.mulatta*	Indian	10	6
**JT27**	M	*M.mulatta*	Chinese	9	6
**JT42**	F	*M.mulatta*	Indian	10	6
**KA87**	F	*M.mulatta*	Indian	9	3
**KA90**	F	*M.mulatta*	Indian	9	6
**KC05**	F	*M.mulatta*	Indian	9	6
**KE20**	F	M.mulatta	Indian	9	4
**KL57**	F	*M.mulatta*	Indian	8	6
**KL96**	F	M.mulatta	Indian	8	2
**KP37**	M	*M.mulatta*	Indian	8	4
**LI33**	F	*M.mulatta*	Indian	6	1
**LL64**	F	*M.mulatta*	Indian	5	2
**LM58**	M	*M.mulatta*	Indian	5	1
**MD12**	F	*M.mulatta*	Indian	4	1
**MD24**	M	*M.mulatta*	Indian	4	1
**Average**				10.7	4.1

Supplement Table 2. Reclassification of individual macaques based on LDA of ECG data.

Actual group	Predicted Chagasic	Predicted Uninfected	Correctly reclassified
Chagasic (N=24)	18	6	18/24 (75%)
Uninfected (N=21)	5	16	16/21 (76%)
Total	23	22	34/45 (75.5%)

Linear discriminant analysis (LDA) is used to predict membership in naturally occurring groups. It answers the question: Can a combination of variables (ECG parameters) be used to predict group membership (Chagasic vs uninfected)? Predicted membership for 45 ECGs is shown, as well as the % of correct classification.

Supplemental Table S3: Individual macaque data

ID	Time of follow-up (months)	Time from infection (months)	dFN/FN	qPCR (parasite eq/ml)	TcI %	TcIV %	TcV %	TcVI %	TcII %	Haplo #	HR	P wave (s)	PR wave (s)	QRS (s)	QT (ms)	RR (s)
IN54	0	63	0.189	7.3723	0.005	0.893	0.102	0.000	0.000	7						
IN54	7	70	0.222	0.0513	0.002	0.070	0.928	0.000	0.000	6	136	0.060	0.120	0.040	260	0.441
IN54	12	82	0.487	0.0250	0.672	0.075	0.089	0.000	0.165	10	151	0.050	0.120	0.040	220	0.397
IN54	13	95	0.112	0.1112	0.600	0.178	0.044	0.000	0.178	9	148	0.040	0.110	0.060	220	0.405
IN54	24	119	0.259	0.3070	0.980	0.014	0.004	0.000	0.002	7	145	0.021	0.077	0.059	229	0.410
IP64	0	84	0.171	0.1539	0.707	0.212	0.081	0.000	0.000	6						
IP64	5	89	0.184	2.3693	0.826	0.006	0.167	0.001	0.000	7	156	0.030	0.080	0.040	140	0.385
IP64	17	106	0.056	0.0947	0.385	0.101	0.000	0.158	0.357	6	177	0.040	0.100	0.040	200	0.339
IP64	27	133	0.057	0.3485	0.489	0.330	0.042	0.088	0.052	10						
JC08	0	58	0.103	12.0794	0.260	0.141	0.025	0.574	0.000	6						
JC08	5	63		2.8619	0.455	0.355	0.118	0.055	0.018	6						
JC08	16	79	0.081	0.3012	0.103	0.712	0.015	0.018	0.152	6	150	0.040	0.140	0.030	260	0.400
JC08	20	99	0.084		0.329	0.205	0.281	0.167	0.019	8						
JC08	29	128	0.104	0.5981	0.287	0.635	0.049	0.000	0.029	6						
JM46	0	45	0.079	22.4506	0.536	0.397	0.040	0.000	0.026	7						
JM46	5	50	0.074	0.1038	0.765	0.193	0.025	0.000	0.017	7						
JM46	9	59	0.049	36.8081	0.999	0.001	0.000	0.000	0.000	8	113	0.040	0.080	0.060	210	0.531
JM46	18	77	0.037		0.998	0.001	0.000	0.001	0.000	12						
JM46	21	98	0.036	4.6839	0.384	0.013	0.005	0.000	0.598	11						
JT42	0	76	0.028	0.0342	0.367	0.447	0.000	0.174	0.012	8						
JT42	5	81	0.038	3.3375	0.984	0.014	0.000	0.001	0.000	7	144	0.030	0.100	0.030	240	0.417
JT42	15	96		0.1698	0.772	0.068	0.000	0.012	0.148	6	165	0.050	0.140	0.040	240	0.364
JT42	17	113	0.036		0.710	0.117	0.000	0.079	0.093	7	158	0.040	0.130	0.030	240	0.380
JT42	27	140	0.039	1.0270	0.663	0.289	0.000	0.000	0.048	4	197	0.038	0.074	0.060	183	0.304
LL64	0	37	0.066	3739.6928	0.538	0.462	0.000	0.000	0.000	2						
LL64	6	43	0.068	43.8183	0.677	0.188	0.090	0.000	0.045	4	187	0.030	0.080	0.050	260	0.321
LL64	14	57	0.056	47.3866	0.638	0.363	0.000	0.000	0.000	2						
LL64			0.043		0.372	0.612	0.016	0.000	0.000	3	161	0.040	0.120	0.060	240	0.373
LL64	22	22	0.059	40.8297	0.397	0.603	0.000	0.000	0.000	2	157	0.040	0.100	0.040	220	0.382
ED57	0	75	0.663	0.4967	0.782	0.218	0.000	0.000	0.000	2						
ED57	6	81	0.639	3.9893	0.182	0.818	0.000	0.000	0.000	2						
ED57	16	97	0.694	4.4615	0.438	0.563	0.000	0.000	0.000	2						
ED57	25	122	0.738	14.5952	0.250	0.750	0.000	0.000	0.000	2	162	0.027	0.067	0.065	202	0.358
JN64	0	35	0.916	3.9518	0.768	0.207	0.000	0.000	0.025	3	153	0.060	0.120	0.040	230	0.392
JN64	18	53	0.670	4.7895	0.222	0.751	0.018	0.000	0.009	4						
JN64	19	72	0.533	0.3279	0.530	0.401	0.069	0.000	0.000	3						
KA90	0	76	0.655	5.2274	0.775	0.163	0.000	0.000	0.062	8						
KA90	5	81	0.663	4.2971	0.943	0.049	0.000	0.000	0.000	7						
KA90	12	93	0.563	14.2547	0.833	0.000	0.111	0.000	0.000	8	167	0.040	0.100	0.060	260	0.359
KA90	25	118	0.635	41.2337	0.286	0.629	0.000	0.000	0.000	3	175	0.040	0.100	0.040	180	0.343

KC05	0	76	0.459	1.5103	1.000	0.000	0.000	0.000	0.000	2						
KC05	5	81	0.635	2.6083	0.403	0.597	0.000	0.000	0.000	3						
KC05	12	93	0.190	3.1033	0.463	0.537	0.000	0.000	0.000	3	202	0.050	0.160	0.040	220	0.297
KC05	25	118	0.076	11.9258	0.909	0.091	0.000	0.000	0.000	3	168	0.040	0.100	0.040	160	0.357
KL57	0	76	0.302	0.5570	0.804	0.178	0.000	0.000	0.018	4						
KL57	5	81	0.338	2.6083	0.982	0.016	0.000	0.002	0.000	4						
KL57	12	93	0.418	5.8137	0.413	0.547	0.000	0.040	0.000	4						
KL57	25	118	0.366	245.6292	0.519	0.444	0.000	0.037	0.000	5						
KP37	0	60	0.29	3.8561	0.985	0.015	0.000	0.000	0.000	5						
KP37	5	65	0.279	44.0813	0.997	0.003	0.000	0.000	0.000	4						
KP37	12	77	0.307	1.0444	0.718	0.282	0.000	0.000	0.000	2	169	0.060	0.120	0.030	240	0.355
KP37	25	102	0.341		0.637	0.363	0.000	0.000	0.000	4	173	0.050	0.100	0.030	220	0.347
GI52	0	45	0.548	7.5863	0.712	0.288	0.000	0.000	0.000	2						
GI52	5	50	0.435	336.3050	0.601	0.399	0.000	0.000	0.000	3						
GI52	25	75	0.462	314.3110	0.471	0.529	0.000	0.000	0.000	2	160	0.040	0.100	0.040	200	0.375
